# Supplementary material for: SOS-mediated prophage induction constrains resistance evolution to DNA-damaging antibiotics
Source: bioRxiv. 2026 Jun 24:2026.06.23.733845. Preprint. [Version 1] doi: 10.64898/2026.06.23.733845 (PMC13320796; doi:10.64898/2026.06.23.733845)
Supplement: Supplement 1 [file media-1.pdf]

Supplementary Table 1 (Table S1). Oligonucleotide primers used in this study.

Overhangs are in lowercase text letters.

Oligo ID	Name	Sequence (5’ → 3’)	Purpose
AZ5	gifsy2NI_fwd	CACACTAGCCTTTGCAAAAAG	Amplification of 734bp region to confirm pTOX allelic exchange deleting *STM1019*
AZ6	gifsy2NI_rev	TGGCGCATAATTGTCAGGTTA	
AZ11	gifsy2_fwd	GAAGTGGCTGAGAGGG	Amplification of 286bp region of gifsy-2 prophage
AZ12	gifsy2_rev	GCTATCATTCCTCCTGCTGA	
AZ16	pSC101_Km_fwd	ACCTGCAGGCATGCAA	Amplification of 4.2kb backbone of pSC101 for Gibson assembly
AZ17	pSC101_Km_rev	GACGTGGTGTTAGCTGTG	
AZ18	STM1019_fwd	TCAAGGATACCAGAGGAGATGT	Amplification of 1170 bp region capturing the DinI-like homolog in 4/74
AZ19	STM1019_rev	TGACTGCAGAATTTCGCTG	
AZ20	STM1019_pSC101_fwd	ACCACAGCTAACACCACGTCTCAAGGATACCAGAGGAGATGT	Amplification of 1170 bp region including STM1019 in 4/74; overhang into pSC101 for Gibson assembly
AZ21	STM1019_pSC101_rev	GAGCTTGCATGCCTGCAGGTtgactgcagaatttcgctg	
AZ22	rbs+mscar_fwd	tttaagaaggagatatacatATGGACTCAACCGAAGC	Amplification of the 730 bp mScarlet-i3 gene block; overhang containing an RBS
AZ23	mscar_rev	TTAGCTGCCTCCGCTT	
AZ24	rbs_cym_fwd	atgtatatctccttcttaaaTACAAACAGACCAGATTGTCTGTTT	Amplification of 4460 bp PCV3 backbone; overhang into a rbs+mscar construct for pAZ3 construction via Gibson assembly
AZ25	mscar_cym_rev	aagcggaggcagctaaCCTGCAGGCATGCAAG	
AZ31	pcym_rev	GTCCCTATCTGCTGCCCTAG	Amplification of 1759 bp region in pAZ3 containing cymR & mscar motifs; overhang into pFCiGrecA for pAZ4 construction via Gibson assembly
AZ34	cymterm_ampR_fwd_redo	tctcatgaccaaaatcccttaacgtgTGCATAATGTGCCTGTCAAATGGA	
AZ38	Sal474_Tn7_external_F	CGATCCACCTGGATTGAGCC	Amplification of the Tn7 region to check for recombination in 4/74
AZ39	Sal474_Tn7_external_R	CTTCTACACCGTTCCGCTGC	
AZ35	pfcigreca3_pcym_	ctagggcagcagatagggacCATTAAA	Amplification of 3375 bp

	fwd	GCTTTCGCGGACTGC	region of pFCiGrecA
AZ40	pfcigrecaORI_rev	GGGCTGTGAGCGCTCTTC	containing precA-GFPova fusion, AmpR, & ORI; overhang into pAZ3 for pAZ4 construction via Gibson assembly
AZ44	10kbhomol_ori_R	TGAGTTTTCGTTCCACTGAGC	Amplification of 845 bp region of pAZ4 containing
AZ45	10kbhomol_ori_F	TTATGCAGGGCTGTGAGCG	the ORI for pAZ5 construction
AZ57	Tn7right_pAZ4ampR_R	tttttgataatctcatgaccaaaatcccttaacgCTTCTGCCTGGTACTACATTGTAC	Amplification of 1077 bp Tn7 right arm from 4/74; overhangs into both the
AZ58	Tn7rightStart_pAZ4ori_F	cttttctacggggtctgacgctcagtggaacgaaaactcaCACGCCGACGAACTGCTGTC	ORI & AmpR of pAZ4 for Gibson assembly of pAZ5
AZ59	Tn7leftend_pAZ4cym_F	tttgcagaatccctgcttcgtccatttgacaggcacaGTCGACAGACGGCCTTTTTTTGT	Amplification of 1041 bp Tn7 left arm from 4/74; overhangs into both the
AZ60-2	Tn7leftStart_pAZ4ori_R	agtcagtgagcgaggaagcggaagagcgctcacagCTGCTGGATGAAGTGGAGGTC	ORI & cymR of pAZ4 for Gibson assembly of pAZ5
SP133	Sal474_Tn7_10kbhomol_recombineer_F	CTGCTGGATGAAGTGGAGGTC	Amplification of 6285 bp region of pAZ5 with 1000 bp of homology to 4/74 Tn7
SP134	Sal474_Tn7_10kbhomol_recombineer_R	CGCCGACGAACTGCTGTC	region for lambda red recombination into 4/74

Supplementary Table 2 (Table S2). Unique mutations of single-step evolved 4/74 prophage(+) & prophage(-)

*indicates mutated genes only seen in tandem with other mutations

Mutated gene(s)	Mutant background(s)	Mutation(s)	Mutation location (CP002487)
**DNA gyrase & topoisomerase**			
gyrA	4/74 prophage(+)<br>4/74 prophage(-)	T → C<br>D87G	2,373,804
	4/74 prophage(-)	C → A<br>D87Y	2,373,805
	4/74 prophage(+)	G → A<br>S83F	2,373,816
	4/74 prophage(-)	G → T<br>A119E	2,373,708
	4/74 prophage(-)	C → T<br>D82N	2,373,820
	4/74 prophage(+)	Δ12bp<br>in frame	2,372,225
gyrB	4/74 prophage(+)	T → G<br>E466D	4,061,187
	4/74 prophage(+)	T → A<br>E466D	4,061,187
	4/74 prophage(+)	G → A<br>S464F	4,061,194
**Efflux pumps & membrane proteins**			
soxR	4/74 prophage(+)	G → C<br>D137H	4,526,081
	4/74 prophage(+)	G → A<br>G145D	4,526,106
	4/74 prophage(+)<br>4/74 prophage(-)	T → C<br>L149P	4,526,118
	4/74 prophage(+)	C → T<br>R20C	4,525,730
	4/74 prophage(+)	Δ27bp	4,526,082

		in frame	
	4/74 prophage(-)	A → G<br>D137G	4,526,082
	4/74 prophage(-)	G → T<br>D137Y	4,526,081
ramR	4/74 prophage(+)	C → A<br>G42V	638,091
	4/74 prophage(+)	A → C<br>L54R	638,055
envZ	4/74 prophage(+)	C → A<br>P248Q	3,680,389
	4/74 prophage(+)	G → T<br>P248Q	3,680,389
*ftsI2**	4/74 prophage(+)<br>4/74 prophage(-)	C → A<br>K3N	1,892,887
*sapB**	4/74 prophage(-)	A → G<br>T137A	1,742,586
**Transcription/ Replication**			
rpoA	4/74 prophage(-)	C → T<br>C269Y	3,604,967
rpoC	4/74 prophage(+)<br>4/74 prophage(-)	G → A<br>G1354E	4,395,405
	4/74 prophage(+)	G → A<br>G1354R	4,395,404
rpoD	4/74 prophage(+)	Δ30bp<br>T526M	3,398,547
	4/74 prophage(+)	A → G<br>N430S	3,398,259
deaD	4/74 prophage(+)	Δ13bp<br>frameshift	3,468,025
*rcsA**	4/74 prophage(+)	G → C<br>R47C	2,025,135
*ygfE**	4/74 prophage(-)	A → C<br>F191V	2,607,731
**Stress response**			

clpP	4/74 prophage(+)	C → T Q87*	502,837
	4/74 prophage(+)	Δ5bp frameshift	503,018
**Metabolism**			
purB	4/74 prophage(+)	G → T L337M	1,276,702
	4/74 prophage(-)	$(ATAAGCG)_{2\to1}$ frameshift	1,276,647
	4/74 prophage(-)	$(ATAAGCG)_{2\to3}$ frameshift	1,276,654
	4/74 prophage(-)	+GATAAGC frameshift	1,276,638
	4/74 prophage(-)	+CGCCACGTT in frame	1,277,146
	4/74 prophage(-)	C → T W43*	1,277,582
	4/74 prophage(-)	T → G Q390P	1,276,542
	4/74 prophage(-)	T → G T170P	1,277,203
	4/74 prophage(-)	+A frameshift	1,276,511:1
	4/74 prophage(-)	G → A Q38*	1,277,599
	4/74 prophage(-)	+G frameshift	1,277,682:1
	4/74 prophage(-)	T → G T409P	1,276,486
	4/74 prophage(-)	Δ10bp frameshift	1,276,635
ribD	4/74 prophage(+)	Δ1bp frameshift	469,772
ribH	4/74 prophage(-)	T → C L151S	470,973
icdA	4/74 prophage(+)	Δ54 bp in frame	1,282,084
*eutC**	4/74 prophage(+)	Δ1bp	2,564,148

		frameshift	
*ackA**	4/74 prophage(+)	A → C<br>T233P	2,446,351
*aceF**	4/74 prophage(+)	Δ1bp<br>S394A<br>frameshift	180,101
*trpD**	4/74 prophage(-)	G → T<br>G356*	1,778,030
*lipB**	4/74 prophage(-)	Δ1bp<br>frameshift	696,930
	4/74 prophage(-)	+AG<br>frameshift	696, 933
**Motility**			
*trmE**	4/74 prophage(-)	Δ13bp<br>frameshift	4,070,004
**Large-scale deletions**			
[mnma], ymfB, STM474_1234, ymfC, icdA, cspH, [envF]	4/74 prophage(+)	Δ6,773bp	1,279,043
[purB], hfld, mnma, ymfB, STM474_1234, ymfC, icdA, STM474_1237, envF, msgA, envE, cspH, pagD, pagC, STM474_t1245, [pliC]	4/74 prophage(+)	Δ14,353bp	1,276,676
[galU], hnr, [rssa]	4/74 prophage(+)	Δ2,847bp	1,804,872
**Intergenic**			
yiaG → / → cspA	4/74 prophage(+)	T → A<br>(+202/-87)	3,857,668
	4/74 prophage(+)	A → G<br>(+176/-113)	3,857,642
*STM474_2610 ←/← guaA**	4/74 prophage(+)	G → T<br>(-106/+51)	2,260,470
*sulA ←/→ yccr**	4/74 prophage(-)	+C<br>(-45/-172)	1,119,250:1

Supplementary Table 3 (Table S3). Ancestral & selected mutant identities

(more detailed information about mutations is available in Supplementary Table 2)

Mutant/sample number	Strain background & replicate	Mutated/affected gene(s)	Mutation	Sequencing coverage	CIP MIC (µg/mL)
4/74 prophage(+)	prophage(+)	n/a	n/a	n/a	0.025
s2	prophage(+) replicate 3	*[purB], hfld, mnma, ymfB, STM474_1234, ymfC, icdA, STM474_1237, envF, msgA, envE, cspH, pagD, pagC, STM474_t1245, [pliC]*	Δ14,353 bp: 1,276,676	34.3x	0.0975
s11	prophage(+) replicate 1	*gyrA*	G → A S83F	264x	0.44
s12	prophage(+) replicate 1	*gyrA*	G → A S83F	232x	0.44
s13	prophage(+) replicate 4	*gyrA*	G → A S83F	69x	0.88
s14	prophage(+) replicate 4	*envZ*	C → A P248Q	268x	0.0625
s15	prophage(+) replicate 4	*icdA; deaD*	Δ54bp: 1,282,084; Δ13bp: 3,468,025	267.9x	0.0625
s16	prophage(+) replicate 4	*ramR; ftsI2*	C → A G42V; C → A K3N	73.5x	0.08
s17	prophage(+) replicate 5	*rpoC; eutC*	G → A G1354R	38.3x	0.22
s18	prophage(+) replicate 5	*gyrA*	T → C D87G	46x	0.44
s19	prophage(+) replicate 5	*gyrA*	T → C D87G	20x	0.44
s99	prophage(+) replicate 3	*gyrA*	T → C D87G	55x	0.22
s102	prophage(+) replicate 2	*clpP*	Δ5bp: 503,018	74.8x	0.0625
s103	prophage(+) replicate 4	*soxR*	G → C D137H	40.4x	0.22
s106	prophage(+) replicate 1	*rpoD; ftsI2*	Δ30bp: 3,398,547; C → A	88x	0.0975

			K3N		
s107	prophage(+) replicate 3	*gyrB*	T → A E466D	21x	0.135
s110	prophage(+) replicate 2	*soxR*	T → C L149P	20x	0.08
s111	prophage(+) replicate 4	*clpP*	C → T Q87*	73.5x	0.08
s114	prophage(+) replicate 1	*gyrB*	T → G E466D	262X	0.135
s115	prophage(+) replicate 3	*[galU], hnr, [rssa]*	Δ2,847 bp: 1,804,872	74.5x	0.0975
s118	prophage(+) replicate 2	*rpoC*	G → A G1366E	67.1x	0.08
s121	prophage(+) replicate 1	*gyrB*	G → A S464F	406.3x	0.22
s122	prophage(+) replicate 4	*ribD*	Δ1bp	174x	0.115
s128	prophage(+) replicate 1	*yiaG → / → cspA*	T → A (+202/-87)	200x	0.135
s129	prophage(+) replicate 4	*yiaG → / → cspA*	A → G (+176/-113)	304x	0.22
s131	prophage(+) replicate 1	*soxR; purB*	Δ27bp: 4,526,082; G → T L377M	296x	0.0975
s132	prophage(+) replicate 1	*gyrA*	T → C D87G	63.8x	0.22
s142	prophage(+) replicate 4	*[mnma], ymfB, STM474_1234, ymfC, icdA, cspH, [envF]*	Δ6,773 bp: 1,279,043	25x	n.d.
s147	prophage(+) replicate 5	*soxR; rcsA*	C → T Q19H; G → C Q47H	34.3x	0.135
s149	prophage(+) replicate 1	*rpoD*	A → G N430S	452x	0.22
s150	prophage(+) replicate 4	*gyrA; soxR; ackA*	Δ12bp: 2,372,225; G→A G145D; A → C T233P	304x	0.22
s153	prophage(+) replicate 5	*soxR; rcsA; aceF*	C → T R20C; G → C Q47H;	191.9x	0.135

			Δ1bp: 180,101		
s154	prophage(+) replicate 4	*ramR; envZ; STM474_2610 ←/← guaA*	A → C L54R; G→ T P248Q; G → T (-106/+51)	368.8x	0.0625
4/74 prophage(-)	prophage(-)	Δ*Gifsy-2* Δ*ST64B* Δ*Gifsy-1* Δ*SopE* *minE→/← rnd* *iolC*	Δ45,596bp: 1,054,761 Δ40,147: 2,039,803 Δ51,187bp: 2,726,265 Δ45,069bp: 2,855,501 G→A (+108/+14) $(G)_{8\rightarrow7}$	n/a	0.035
s3	prophage(-) replicate 1	*gyrA*	G → A D82N	147.9x	0.22
s4	prophage(-) replicate 4	*purB*	T → G T170P	274x	0.0975
s20	prophage(-) replicate 1	*gyrA*	C → A D87Y	27.4x	0.22
s21	prophage(-) replicate 1	*purB; ftsI2*	$(ATAAGCG)_{2\rightarrow1}$; C → A K3N	97.9x	0.0975
s22	prophage(-) replicate 1	*purB; ftsI2*	+GATAAGC; C → A K3N	44.8x	0.0975
s25	prophage(-) replicate 1	*gyrA*	C → T D82N	28.7x	0.22
s26	prophage(-) replicate 1	*rpoC*	G → A G1354Q	57.9x	0.135
s27	prophage(-) replicate 1	*purB; ftsI2*	$(ATAAGCG)_{2\rightarrow1}$; C → A K3N	80.6x	0.0975
s28	prophage(-) replicate 2	*purB; sapB; sulA ←/→ yccR*	T → G T409P; A → G T137A; +C (-45/-172)	380x	0.0975
s30	prophage(-) replicate 2	*purB*	+GATAAGC	71.2x	0.0975
s31	prophage(-) replicate 2	*purB; trpD*	G → A Q38* G → T G356*	20x	0.0975

s32	prophage(-) replicate 2	*purB*	C → T W43*	60.3x	0.0975
s33	prophage(-) replicate 2	*purB*	+GATAAGC	23.9x	0.0975
s34	prophage(-) replicate 2	*purB*	T → G T409P	299x	0.0975
s35	prophage(-) replicate 2	*purB; ftsI2*	T → G Q390P; C → A K3N	37.9x	0.0975
s36	prophage(-) replicate 3	*gyrA*	G → T A119E	37.2x	0.22
s37	prophage(-) replicate 3	*purB*	+GATAAGC	49.2x	0.115
s38	prophage(-) replicate 3	*purB*	+GATAAGC	53.4x	0.0975
s40	prophage(-) replicate 3	*gyrA*	T → C D87G	57.2x	0.22
s41	prophage(-) replicate 3	*purB; ribH; trmE*	Δ10bp: 1,276,365; T → C L151S; Δ13bp: 4,070,004	218x	0.08
s42	prophage(-) replicate 3	*soxR*	T → C L149P	43.6x	0.22
s43	prophage(-) replicate 4	*soxR*	G → T D137Y	47.5x	0.135
s44	prophage(-) replicate 4	*purB*	+G	315x	0.0625
s45	prophage(-) replicate 4	*soxR; yfgE*	G → T D137Y; A → C F191V	165x	0.22
s47	prophage(-) replicate 4	*soxR*	A → G D137G	351x	0.135
s48	prophage(-) replicate 4	*soxR*	G → T D137Y	51.2x	0.22
s49	prophage(-) replicate 4	*purB*	+A	91.8x	0.08
s50	prophage(-) replicate 5	*purB; ftsI2*	+GATAAGC; C → A K3N	71.5x	0.115
s51	prophage(-) replicate 5	*purB*	+GATAAGC	93.6x	0.08

s140	prophage(-) replicate 3	*soxR*	T → C L149P	56.7x	n.d.
s143	prophage(-) replicate 5	*rpoA*	C → T C269Y	25x	0.135
s144	prophage(-) replicate 5	*purB*	+CGCCACGTT	266x	0.08
s156	prophage(-) replicate 5	*purB; lipP*	+ATAAGCG; Δ1bp: 696,930, +AG	89.4x	0.44

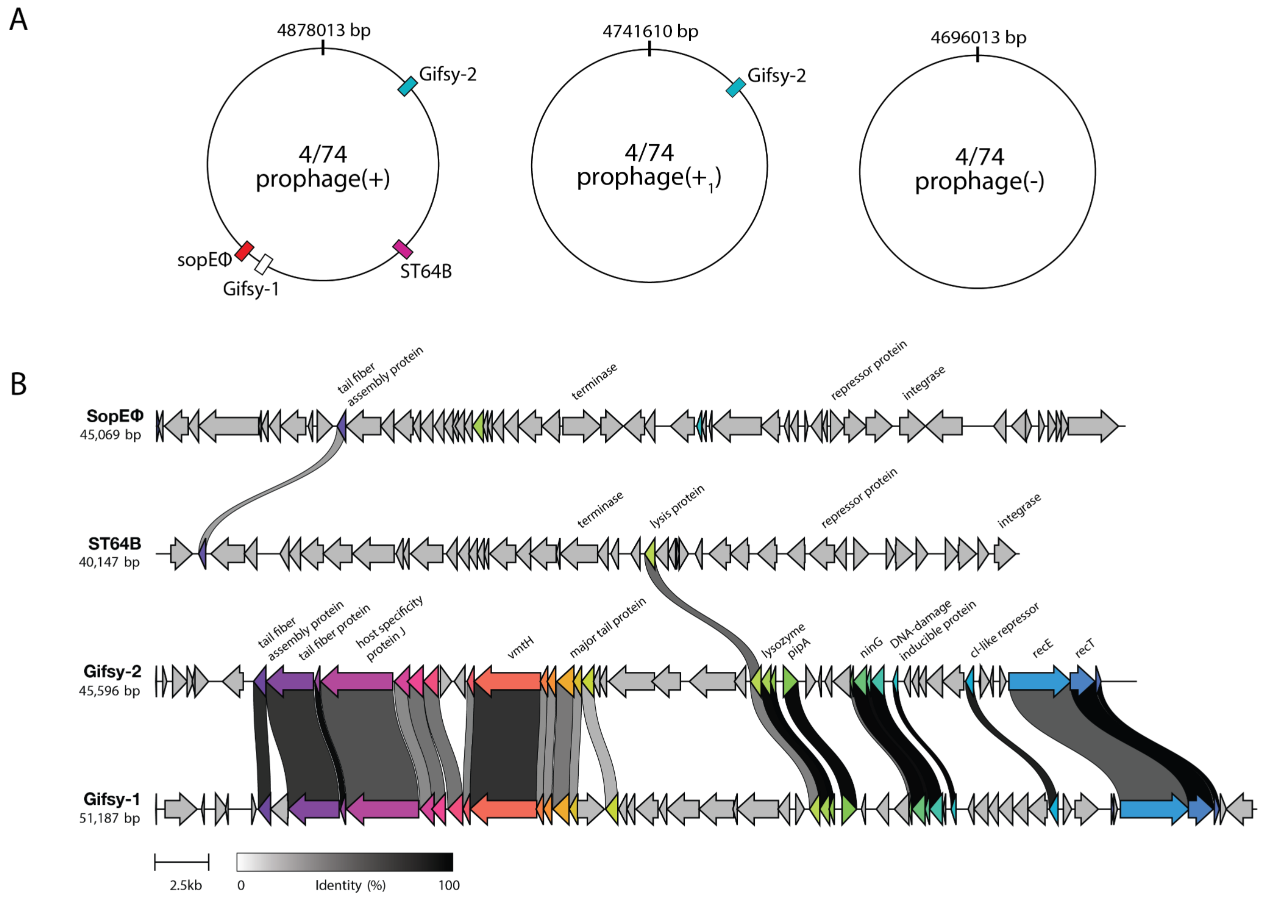

Supplementary Figure 1 (S1). *Salmonella* Typhimurium 4/74 prophage composition & synteny.

Prophage regions were identified within each variant of 4/74 and were internally compared to assess similarity. **A.** Schematic representation of prophage composition across *S.* Typhimurium variants used in this study, highlighting the location of each mobile genetic element. **B.** Comparative synteny analysis generated using clinker, showing homologous protein clusters and gene similarity across each integrated prophage. Genes are represented as directional arrows with homologous genes grouped by shared sequence similarity and colored accordingly.

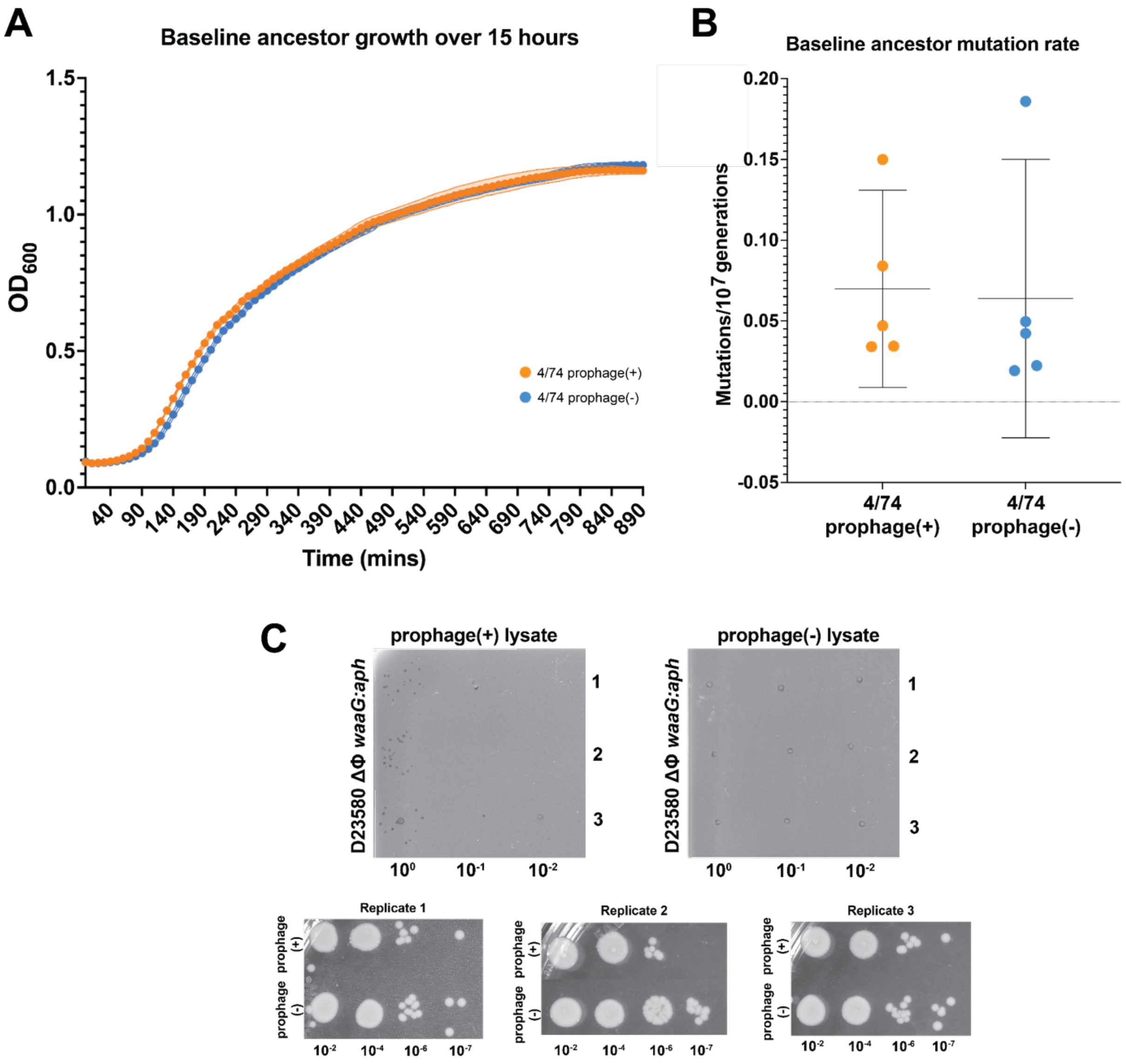

Supplementary Figure 2 (S2). There are no prophage-dependent differences across growth and spontaneous mutation rate for ancestral *Salmonella* Typhimurium.

A. Growth curves of lysogenic 4/74 prophage(+) and isogenic 4/74 prophage(-). Colored points represent OD600 measurements collected every 10 min for 13 h across four biological replicates. No measurable differences in growth rate were observed between backgrounds. **B.** Fluctuation assay calculations of spontaneous mutation rates per 10^7 bacterial generations. Each point represents the estimated mutation rate (see Methods) for an individual biological replicate colored by ancestral background. There was no measurable difference in mutation rate between strains. **C.** Representative plaque assays and corresponding colony counts used to quantify spontaneous prophage induction following 24 h incubation. Filtered lysates were spotted onto lawns of indicator strain *Salmonella enterica* serovar Typhimurium strain D23580ΔΦ *waaG:aph*. Plaque- and colony-forming units were used to calculate a spontaneous induction rate of 6 × 10^-6 PFU/CFU for 4/74 prophage(+).

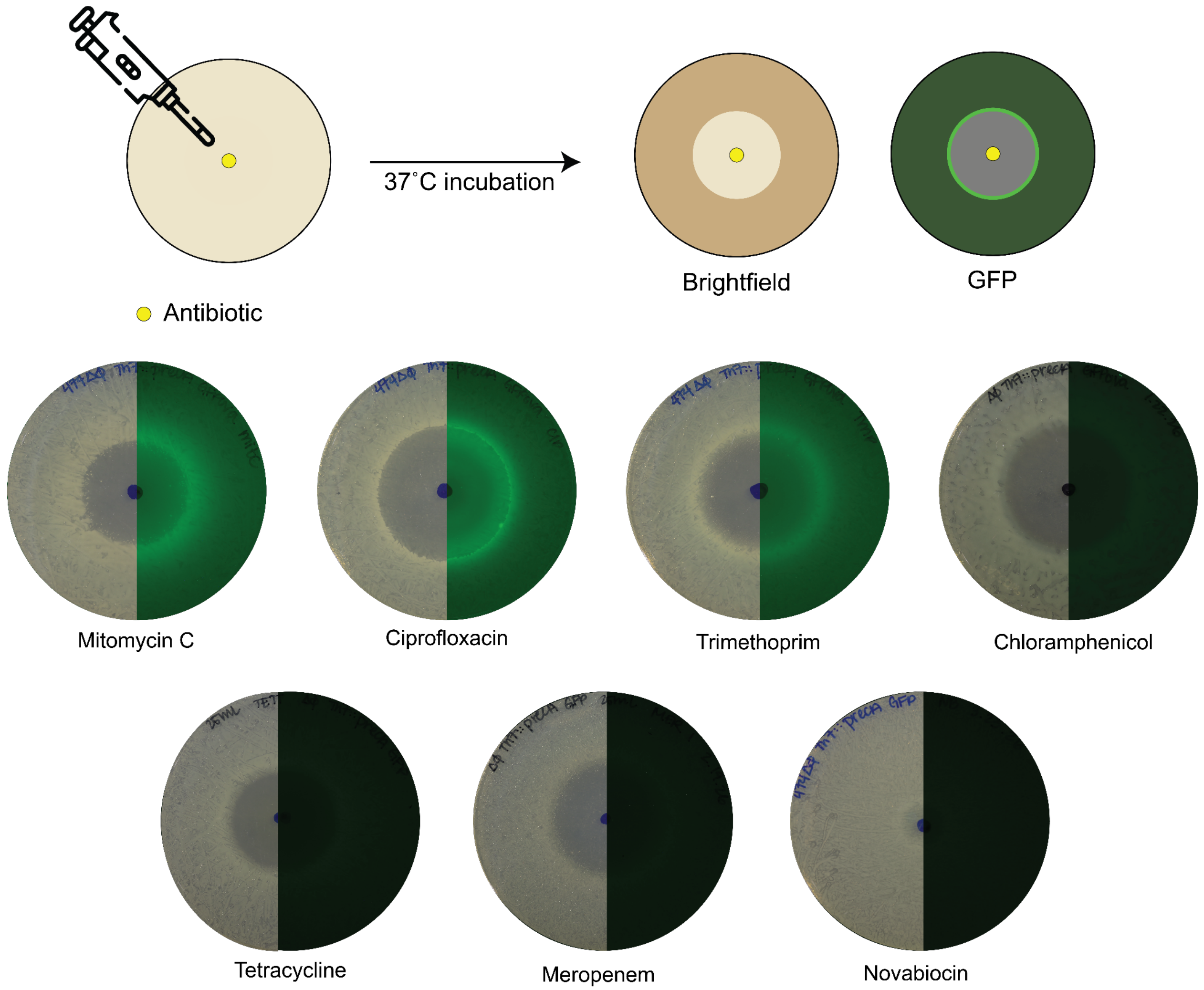

Supplementary Figure 3 (S3). SOS response induction is dependent on antibiotic class and mechanism of action.

Drop assays demonstrating SOS response activation through *precA* reporter expression. Concentrated antibiotics (5 µL) were applied to the center of 25 mL 2% LB agar plates and allowed to diffuse for >5 h prior to inoculation with bacterial lawns carrying a chromosomal *precA*-GFPova reporter. Plate images are shown as half brightfield and half GFP fluorescence. SOS activation is indicated by GFP fluorescence surrounding and extending beyond the zone of inhibition.

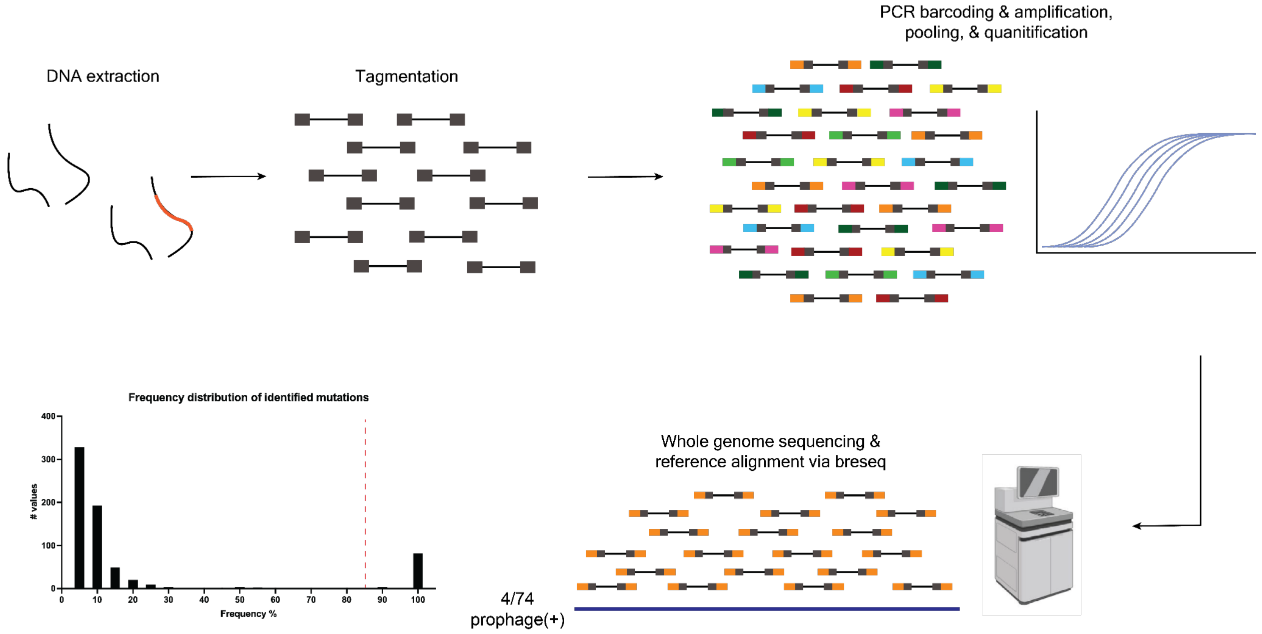

Supplementary Figure 4 (S4). Whole genome sequencing alignment via *breseq* provides high coverage mapping to reference genomes

Workflow for whole-genome sequencing and analysis of CIP-resistant isolates. Genomic DNA was extracted from clonal overnight cultures and tagmented as described by Baym et al. Unique dual 8 bp barcodes were added to tagged DNA fragments by PCR using custom primers (Supplementary Table 1). Amplicons were pooled, quantified by qPCR, and sequenced at the Harvard Bauer Core Facility. Sequencing reads were quality controlled using FastQC, preprocessed with Trimmomatic, aligned using minimap2 and SAMtools, and analyzed with *breseq* against the 4/74 WT reference genome. Because isolates were clonal, mutation calling thresholds were set at 85% allele frequency.

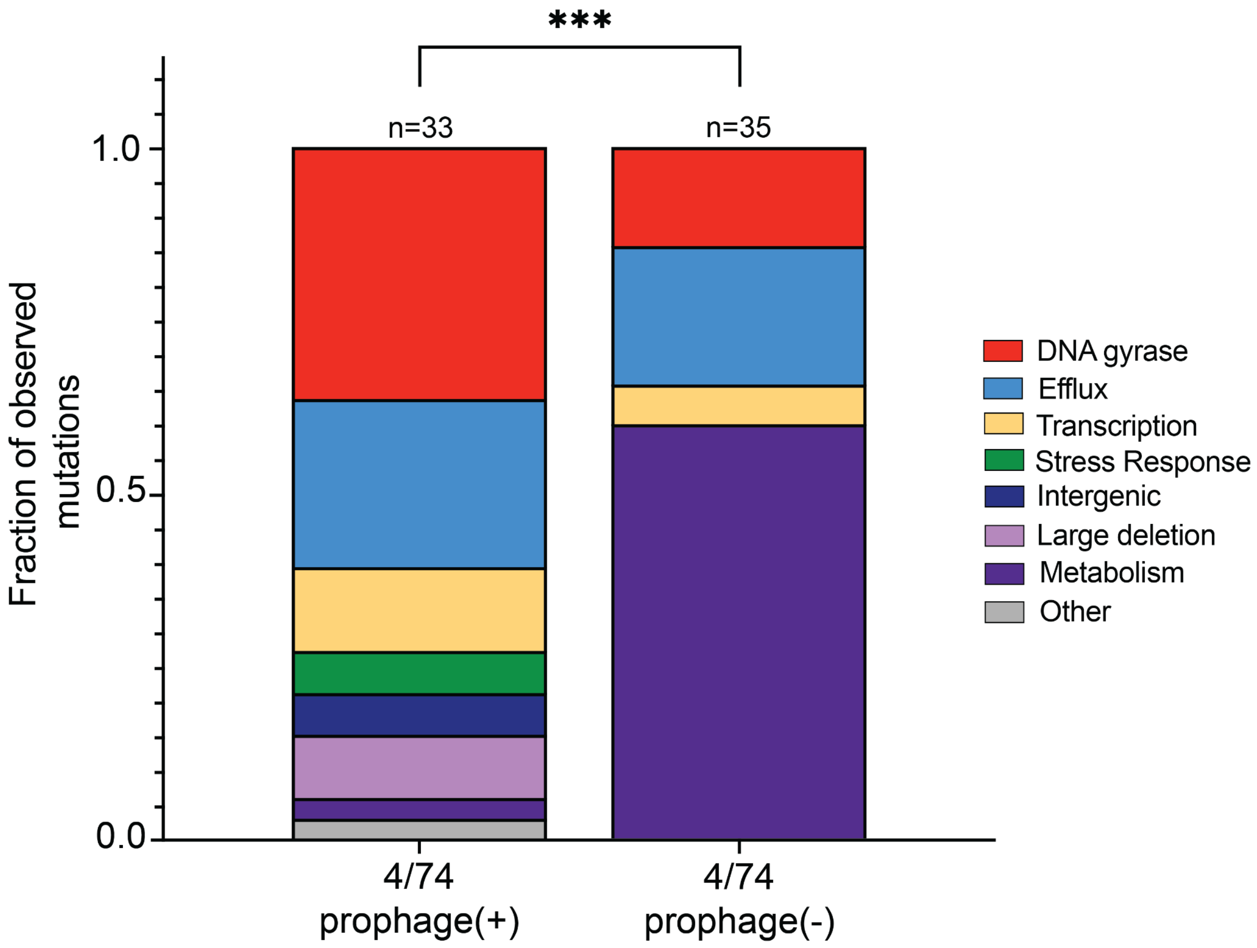

Supplementary Figure 5 (S5). Absolute mutated pathway distributions also differ by prophage carriage.

Bar plots showing the distribution of overall observed pathways/categories represented by genes carrying resistance-conferring mutations in 4/74 prophage(+) and prophage(-) backgrounds. Each colored segment indicates the fraction of total observed mutations assigned to a given host function pathway. Pathway distributions differed significantly between ancestral backgrounds (permutation chi-square test, 10,000 label shuffles, $P<0.0001$). As in Figure 3, quinolone resistance pathways (DNA gyrase and efflux pump) were mutated in both backgrounds, but comprised a larger fraction of mutations in prophage(+), but this enrichment was not significant (Monte Carlo permutation test; Fisher's exact test, n.s.).

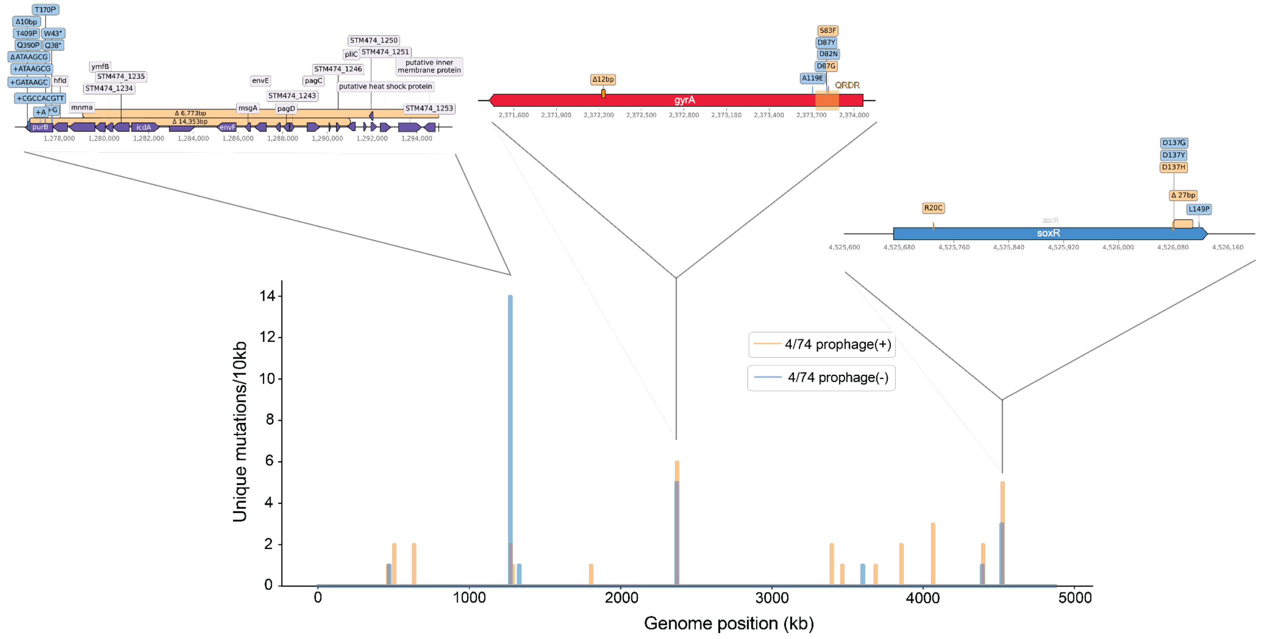

Supplementary Figure 6 (S6). Distributions of mutated residues differ by prophage carriage in mutation location and type.

Genomic distribution of unique resistance-associated mutations in prophage(+) and prophage(-) CIP-resistant mutants. Vertical lines within 10 kb genomic bins indicate the presence of one or more mutations. Insets highlight regions of elevated mutational variability, including *purB*, *gyrA*, and *soxR*. Annotated genes are shown as solid arrows with labels indicated on or above (gray box) each feature. Mutations are represented by colored markers corresponding to prophage(+) and prophage(-) mutant backgrounds (orange and blue respectively).

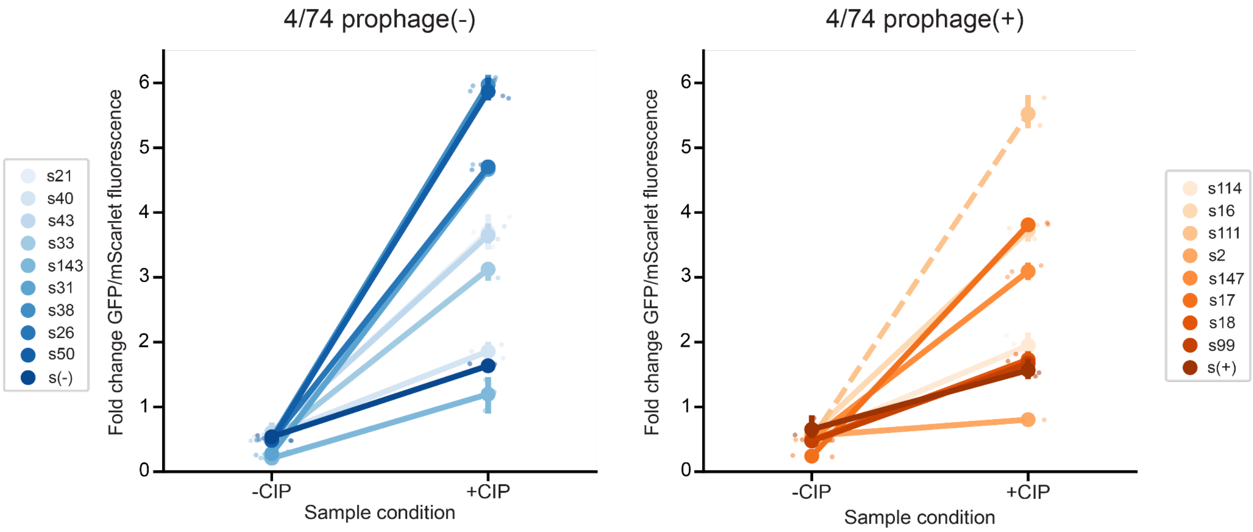

Supplementary Figure 7 (S7). SOS response induction is dampened in lysogenic mutants, while non-lysogen metabolism mutants maintain high SOS induction.
Mutant-specific cell-size (mScarlet-i3) normalized precA-GFP expression in the absence (−CIP) and presence (+CIP) of sub-inhibitory ciprofloxacin for prophage(−) (blue) and prophage(+) (orange) resistant mutants. Lines connect measurements from the same mutant across conditions, illustrating changes in SOS induction following ciprofloxacin exposure. Shades represent individual mutants (identities listed in Table S3). Small points indicate biological replicates (n = 3), and large points indicate the mean value for each mutant. Under sub-inhibitory ciprofloxacin treatment, prophage(+) mutants exhibited lower SOS activation and a reduced fold change in GFP expression compared with prophage(−) mutants (Mann–Whitney U test, P = 0.0144). Sample 111 (s111; dashed line) carries a mutation in *clpP* and was considered incompatible with the precA-GFP reporter, as disruption of *clpP* is predicted to impair SOS signaling downstream of recA activation.

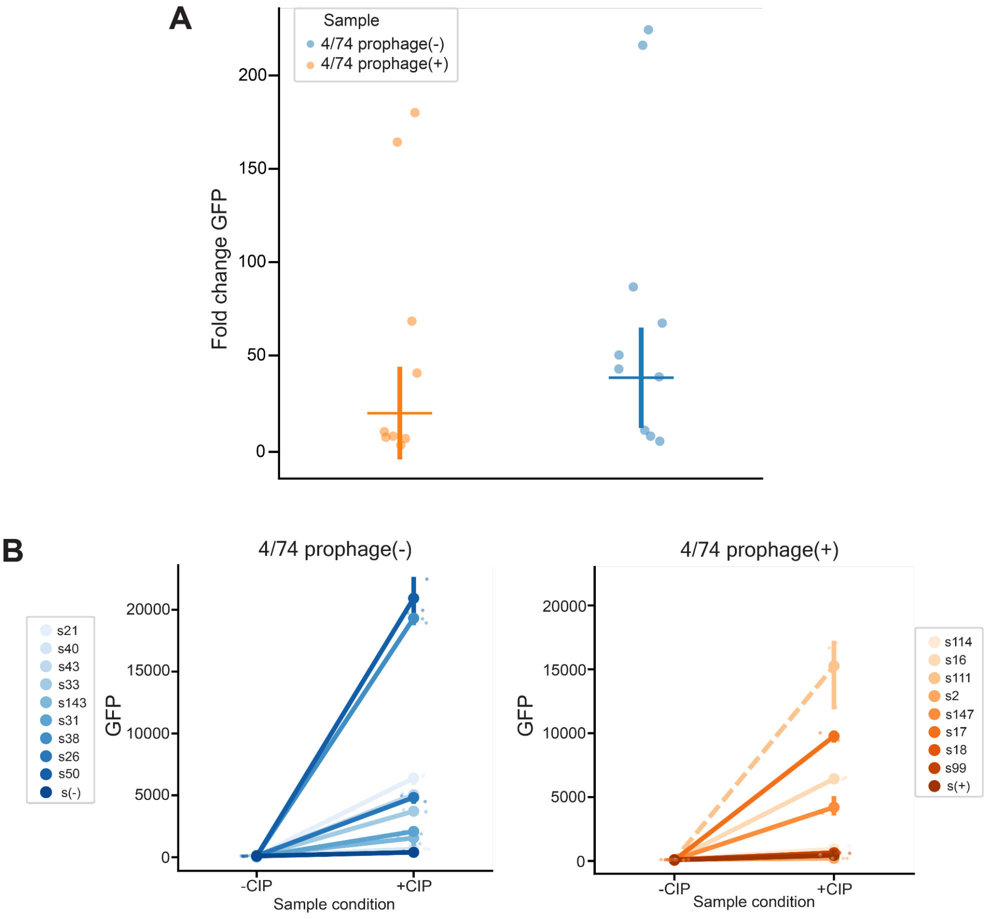

Supplementary Figure 8 (S8). Dampened SOS response of CIP-resistant lysogens is consistent with or without cell size normalization.

A. Fold change in precA-GFP expression across −CIP and +CIP (sub-inhibitory) conditions for prophage(-) (blue) and prophage(+) (orange) resistant mutants. Each point represents a single mutant's mean GFP expression for 3 biological replicates. Prophage(+) mutants showed significantly lower average induction than prophage(-) mutants, matching the normalized results (Fig. S7, Mann–Whitney U test, $P = 0.03245$). **B.** Mutant-specific precA-GFP expression in the absence (−CIP) and presence (+CIP) of sub-inhibitory ciprofloxacin for prophage(−) (blue) and prophage(+) (orange) resistant mutants. Lines connect measurements from the same mutant across conditions, illustrating changes in SOS induction following ciprofloxacin exposure. Shades represent individual mutants (identities listed in Table S3). Small points indicate biological replicates ($n = 3$), and large points indicate the mean value for each mutant. Under sub-inhibitory ciprofloxacin treatment, prophage(+) mutants exhibited lower SOS activation and a reduced fold change in GFP expression compared with prophage(−) mutants, matching normalized results (Fig. S7). As under normalized conditions, sample 111 (s111; dashed line) carries a mutation in *clpP* and was considered incompatible with the precA-GFP reporter, as disruption of *clpP* is predicted to impair SOS signaling downstream of recA activation.

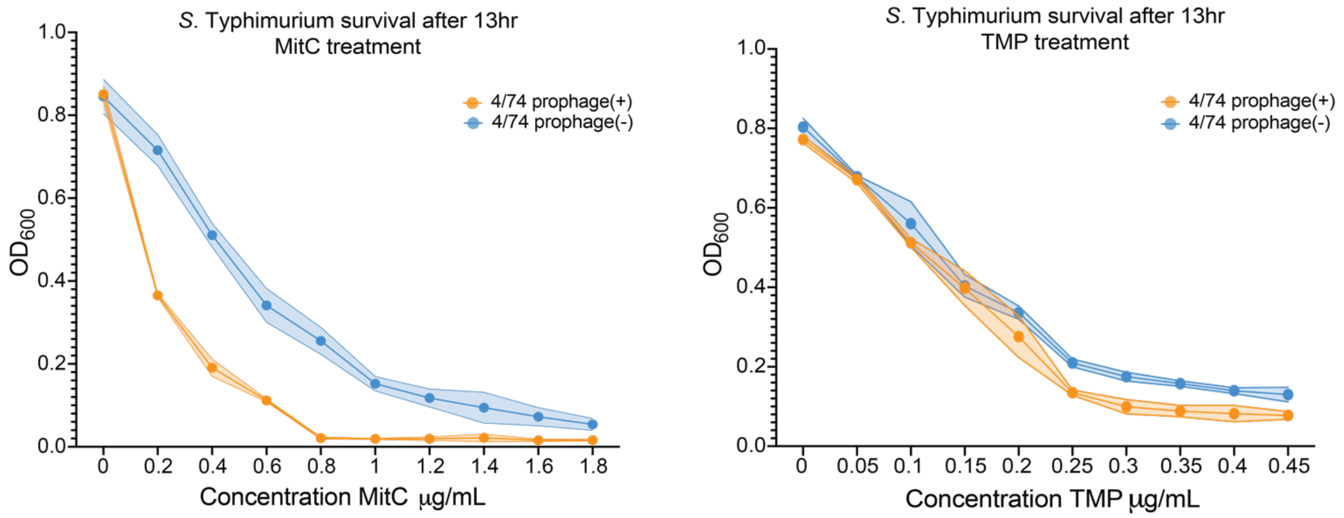

Supplementary Figure 9 (S9).

OD_{600} values of prophage(+) and prophage(-) strains showing survival after 13 h growth in increasing concentrations of antibiotic treatment (MitC & TMP, x-axis) as a measure of sensitivity. Points represent mean ± SD of biological replicates. Survival of prophage(+) samples was significantly lower than the non-lysogen at every MitC treatment (multiple *t*-tests with FDR correction, adjusted $P < 0.001$). TMP treatment yielded little to no differences in antibiotic sensitivity across strains. Although significant at high TMP concentrations ($P < 0.01$), these differing values were close to the limit of detection for the plate reader.
